# Mandibular third molar extraction: perceived surgical difficulty in relation to professional training

**DOI:** 10.1186/s12903-023-03131-7

**Published:** 2023-07-14

**Authors:** María Isabel Sánchez Jorge, Rosa Acevedo Ocaña, Carolina Valle Rodríguez, Barbara Peyró Fernández-Montes, Cristina Rico-Romano, Santiago Bazal-Bonelli, Luis Sánchez-Labrador, Jorge Cortés-Bretón Brinkmann

**Affiliations:** 1 Department of Oral Surgery and Oral Implantology, Faculty of Dentistry, University Alfonso X El Sabio, Madrid, Spain; 2 Doctoral Programme in Health Sciences, University of Alcala de Henares, Madrid, Spain; 3 Doctoral Programme in Surgery and Odontostomatology, University of Salamanca, Salamanca, Spain; 4 Department of Conservative and Aesthetics Dentistry, Faculty of Dentistry, University Alfonso X El Sabio, Madrid, Spain; 5 Department of Dental Clinical Specialties, School of Dentistry, Complutense University of Madrid, Plaza Ramon Y Cajal S/N, 28040 Madrid, Spain; 6 Surgical and Implant Therapies in the Oral Cavity Research Group, Complutense University of Madrid, Madrid, Spain

**Keywords:** Third molar, Mandibular, Extraction, Perceived difficulty, Professional training

## Abstract

**Background:**

Establishing the level of surgical difficulty pre-operatively is an essential step in ensuring correct treatment planning. This study set out to determine whether the knowledge and experience acquired by dentists who had received different levels of training influenced, firstly, the perceived levels of difficulty of a variety of cases of mandibular third molar (MTM) extraction and, secondly, the perceived difficulty deriving from a series of factors (patient-related factors, anatomical and radiographic factors, operative factors).

**Methods:**

This cross-sectional, descriptive, observational study took the form of a survey. Using a visual analog scale (VAS), participants evaluated both the perceived difficulty of 30 cases of MTM extraction described by means of digital panoramic radiographs and the perceived difficulty deriving from a series of factors conditioning MTM extraction. The results underwent statistical analysis with SPSS Statistics 28.0 software. Non-parametric tests (Mann Whitney test for independent samples and the Kruskal–Wallis test) were applied.

**Results:**

A total of 389 surveys were available for analysis. Dental practioners with no surgical training saw the intervention as presenting greater difficulty. Professionals with postgraduate training in oral surgery considered patient-related factors more important than operative factors, in contrast to dentists who had not received oral surgery training.

**Conclusions:**

Dental training has a signficant influence on the perceived difficulty of MTM extraction and also affects opinions about which factors have greater or lesser influence on surgical difficulty.

**Supplementary Information:**

The online version contains supplementary material available at 10.1186/s12903-023-03131-7.

## Background

Mandibular third molar (MTM) extraction is one of the most common types of oral surgery [1]. The intervention is laborious, intricate, fairly challenging, and calls for correct, rigorous, and measured procedures.

Given the wide range of positions and situations that mandibular third molars can present, the intervention cannot be established or described as a single type. For this reason, researchers have attempted to develop classifications related to levels of difficulty, aimed at guiding clinicians performing the extraction procedure. In 1976, MacGregor was the first to design a classification system, elaborating a multivariant model based on radiographic findings [2]. His observations became the basis for subsequent proposals. Among the most widely used classifications, those by Winter [3], Pell and Gregory [4], and Pederson [5], are considered classics. Nevertheless, other dental professionals have created their own scales of difficulty based on specific variables [6–10]. Initially, authors analyzed radiographic variables in orthopantomographs such as: the size and shape of the dental crown; number size, and curvature of the roots; the impaction’s position and situation; presence or absence of periodontal ligament; relationships with adjacent structures [2–6, 11, 12]. But later, researchers began to take a broader range of variables into consideration, incorporating them into their difficulty scales. These included variables related to the patient (age, sex, ethnicity, body mass index, mouth opening) and operative variables (need for flap raising, for ostectomy, for odontosection, the clinician’s experience) [6, 13–22].

Establishing the level of surgical difficulty pre-operatively is an essential step in ensuring correct treatment planning. This will allow clinicians to prepare the appropriate equipment and materials, to decide on the right point of surgical access, the right technique, the type of anesthesia, and to determine whether or not the individual clinician’s capabilities are up to the extraction in question. These measures will lead to an intervention of lower risk and fewer intra- and post-operative complications [23, 24].

Various researchers have assessed dentists’ capacity for predicting surgical difficulty, with widely varying results [15, 23, 25–29]. The present work set out to analyze the extent to which perceptions of surgical difficulty of the said intervention vary in relation to professional training. To the authors' knowledge, no previous published studies have investigated the relationships between perceived difficulty and professional training.

To do this, we felt it would be useful and helpful to determine (by means of a survey) whether the knowledge and experience acquired by dentists with different levels of training influenced, firstly, the perceived levels of difficulty of a variety of cases of mandibular third molar (MTM) extraction and, secondly, the perceived influence of a series of factors (patient-related factors, anatomical and radiographic factors and operative factors) on difficulty.

## Methods

### Study design, survey validation, and sample size

This cross-sectional, descriptive, observational study took the form of a survey. The survey was formulated with EUSurvey software, the European Commission’s official tool for conducting surveys.

The survey was designed by a team of oral surgery specialists and was informed by the available literature addressing the difficulty levels and factors influencing MTM extraction. Before delivery to participants, the team presented the survey to four experts in oral surgery who did not participate in the study. They were asked to analyze the survey questions in terms of clarity and ease of comprehension. Their corrections and suggestions were introduced, giving the survey its final form.

Lastly, the Intraclass Correlation Coefficient (ICC) and Cronbach's alpha were calculated to evaluate variability in repetition of the survey, and reliability of the measurement scale, respectively.

Invitations were sent to potential participants, explaining the objectives and reasons for the survey and its approximate duration. The survey was made available online to those dental professionals willing to participate.

The participants had received varying types of training and acquired different levels of experience. They included dental undergraduates, dental graduates without post-graduate qualifications, graduates with postgraduate qualifications in some specialization, and graduates with postgraduate qualifications in oral surgery. A link to the survey was disseminated by e-mail and via Whatsapp to current students, former students, teaching staff, work colleagues, and research colleagues at a range of Higher Education Centres in Madrid (Spain) that offer different types of dental training and different qualifications.

Before completing the survey, participants gave their informed consent to take part. In turn, they were provided with a guarantee of anonymity and data privacy.

The survey consisted firstly of a series of items related to the participants’ demographic and academic data (age, sex, academic situation, year of graduation, post-graduate programs completed (if any), type of post-graduate program(s), duration of post-graduate program(s)).

Secondly, using visual analogue scales (VAS), the participants assessed the importance of a series of patient-related factors (sex, age, ethnicity, mouth opening, body mass index), anatomical and radiographic factors (root morphology, root curvature, lower third molar position, lower third molar situation, depth of impaction, impaction of lower third molar in the ascending ramus, proximity of the lower third molar to the inferior alveolar nerve), and operative factors (anesthetic technique: local vs. general anesthesia; need for flap lifting; need for ostectomy; need for odontosection; clinician’s experience), which could influence the surgical difficulty of lower third molar extraction. On the scale, “0” represented “minimum influence” on surgical difficulty and “10” represented “maximum influence.”

Lastly, participants assessed (using VAS) the levels of difficulty of 30 cases of MTM extraction described by high quality digital panoramic radiographs, all of which showed at least one lower third molar. On the scale of 0 to 10, “0” represented “no difficulty” and “10” represented “maximum difficulty.” The radiographs were selected from those pertaining to patients treated on the University’s Master’s Program in Oral Surgery and Implantology, who gave their consent for their radiographs to be used anonymously in the study.

The sample size was determined according to a previous, similar study [15] and one of the main variables: age. A preliminary sample size calculation was made using specialized software (G*Power 3.1.9.4). The calculation revealed a total sample size of 11 participants per group (dental undergraduates, dental graduates without post-graduate qualifications, graduates with postgraduate qualifications in some specialization, and graduates with postgraduate qualifications in oral surgery) with an effect size of 21,1 at 0.8 power and a significance level of 0.05.

### Study approval

The study was approved by the Committee for Ethics in Research at the San Carlos Hospital, Madrid, Spain (C.I. 22/135-E, 14th March 2022), and followed the ethical guidelines established in the Declaration of Helsinki by the World Medical Association.

### Data collection and statistical analysis

The results of the survey were entered and stored on a EUSurvey spreadsheet and later underwent statistical analysis with SPSS* Statistics 28.0 software (SPSS® inc, Chicago IL, USA).

Statistical analysis was conducted at the University’s Data Processing Center. Firstly, a descriptive study of frequencies was performed, calculating means, median values, standard deviations, and ranges. Secondly, data were analyzed with inferential statistics with a 95% confidence Interval, and so a significance level of *p* < 0.05.

Applying the Kolmogorov–Smirnov test, it was found that data did not display normal distribution, and so non-parametric tests were used: the Mann Whitney test for independent samples (two comparative groups) and the Kruskal–Wallis test (more than two comparative groups). Whenever the Kruskal–Wallis test indicated significant differences, paired comparisons with Bonferroni corrections were made.

## Results

### Subject characteristics

The survey was sent to 670 people; 390 of whom (58.2%) completed the survey online. One participant later refused to provide informed consent to take part, so 389 (58.1%) surveys were available for analysis. Distribution of the sample in terms of age, sex, and academic situation is shown in Table 1.

**Table 1:** Sample distribution by age, sex, and academic situation

**Age group (years)**		Frequency (nº)	Percent (%)
Valid	18–22	45	11,6
	23–27	44	11,3
	28–30	32	8,2
	31–35	51	13,1
	36–45	141	36,2
	≥ 46	76	19,6
	Total	389	100,0
**Gender**		Frequency (nº)	Percent (%)
Valid	Female	222	57,1
	Male	165	42,4
	Other	2	0,5
	Total	389	100,0
**Academic situation**		Frequency (nº)	Percent (%)
Valid	Graduate dentists	328	84,3
	Undergraduated students	61	15,7
	Total	389	100,0

15.7% of participants were undergraduate students; all of them were fifth (final) year students. The remaining 84.3% had completed the degree course in dentistry.

Graduate dentists were asked how long ago they had completed training, as well as whether they had completed any post-graduate program(s). Those who had post-graduate qualifications were asked about the type of course completed (oral surgery, implant dentistry, periodontics, orthodontics, prosthetics, endodontics, or others). Those who had completed post-graduate programs in oral surgery or who were currently enrolled on one were asked to state the program’s duration.

Participants were asked whether they were aware of the factors that exert an influence on the surgical difficulty of lower third molar extraction and whether they had received information about such factors. 50.8% of dental undergraduates stated that they were unaware of these factors compared with 3.6% of graduate dentists who lacked this information. Considering each group in isolation, 7.4% of graduate dentists without specialized training in oral surgery stated that they were unaware of these factors; only 0.5% of dentists with postgraduate training in oral surgery said they were unaware of them, all of these having received training via a modular course with non-weekly attendance.

### Survey validation

The survey was repeated one week later by a large and representative sample of participants (12.05%) in order to evaluate the variability of the survey. The ICC was calculated for every participant determining excellent concordance in all cases (values > 0.8), confirming the survey’s validity. In addition, Cronbach's alpha was calculated to measure the reliability of the measurement scale, obtaining a value of 0.83, which guaranteed its reliability.

To provide a detailed and comprehensible analysis of the results, three comparative groups were established. (Table 2).

**Table 2:** Comparative groups

GROUP 1		Frequency (nº)	Percent (%)	Valid Percent (%)
Valid	Dental undergraduates	61	15,7	15,7
	Graduate dentists	328	84,3	84,3
	Total	389	100,0	100,0
GROUP 2		Frequency (nº)	Percent (%)	Valid Percent (%)
Valid	Graduate dentists without post-graduate training	33	8,5	10,06
	Graduate dentists with postgraduate training in other specializations than oral surgery	115	29,5	35,06
	Graduate dentists with post-graduate training in oral surgery	180	46,3	54,88
	Total	328	84,3	100,0
Missing	System	61	15,7	
Total		389	100,0	
GROUP 3		Frequency (nº)	Percent (%)	Valid Percent (%)
Valid	Graduate dentists who completed post-graduate programs in oral surgery of **3 years**	58	14,9	37,4
	Graduate dentists who completed post-graduate programs in oral surgery of **2 years**	77	19,8	49,7
	Graduate dentists who completed post-graduate programs in oral surgery of **1 year**	11	2,8	7,1
	Graduate dentists who completed post-graduate programs in oral surgery by **modular** course with non-weekly attendance	9	2,3	5,8
	Total	155	39,8	100,0
Missing	System	234	60,2	
Total		389	100,0	

### GROUP 1 (dental undergraduates vs. graduate dentists)

Comparing the responses given by undergraduates and graduate dentists regarding the influence of different factors on surgical difficulty, significant differences were found (applying the Mann–Whitney U test for independent samples) for 11 out of the 17 factors investigated. No statistically significant differences were found for the sex of the patient, whereby both undergraduate and graduate dentists awarded this factor little importance (mean 2.9 out of 10). Nor were significant differences identified for the factors root morphology or curvature, impaction depth, impaction in the ramus, or clinical experience. These items were awarded values of over 8.5, and so were seen as exerting an important influence on surgical difficulty.

Regarding the 11 factors that did present statistically significant differences between these groups, graduate dentists awarded higher scores than undergraduates to only three (ethnicity, mouth opening, and body mass index), while undergraduates gave higher scores to the other eight (age, position, situation, proximity to the alveolar nerve, anesthetic technique, need for flap raising, ostectomy, and odontosection). So, undergraduates gave lower scores to patient-related factors and higher scores to anatomical, radiographic and operative factors. The groups also differed in the factor allotted the greatest importance, whereby undergraduates considered proximity to the inferior alveolar nerve the most influential factor, while graduates considered the clinician’s experience the most influential.

**Fig. 1:**
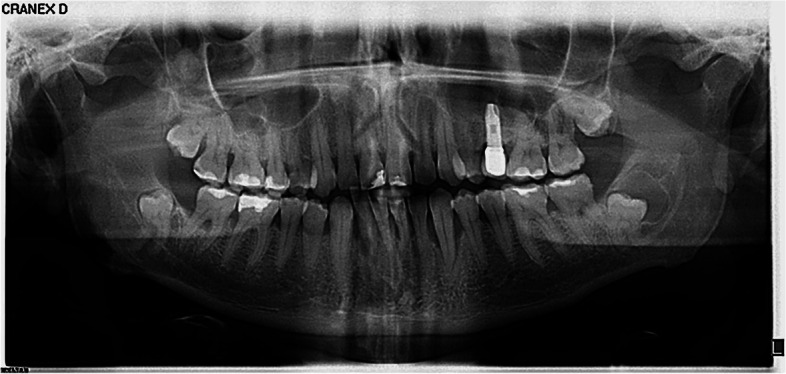
Panoramic radiograph number 8 used in the survey for evaluation of MTM 48

Comparing the difficulty values (Mann–Whitney U test) awarded to each of the 30 cases of mandibular third molars described in panoramic radiographs, significant differences (*p* < 0.05) were found for seven out of the 30 cases. Of these seven, undergraduates saw two cases as presenting more difficulty (both of them with molars in horizontal positions), while in the other five cases (with lower third molars in vertical positions) graduate dentists awarded higher scores. (Fig. 1 and Table 3).

**Table 3:** Difficulty values given by dental undergraduates and graduate dentists to MTM 48 of radiograph 8 shown in the survey. U Mann–Whitney test

			Valid N	Mean	Standard Deviation	Median	Percentile 25	Percentile 75	Minimum	Maximum
8. Extraction of 4.8	Group 1	Dental undergraduates	61	8,46	1,75	9,00	8,00	10,00	2,0	10,0
		Graduate dentists	328	9,40	1,05	10,00	9,00	10,00	0,0	10,0
		Total	389	9,25	1,23	10,00	9,00	10,00	0,0	10,0
**Independent-Samples Mann–Whitney U Test Summary**										
Total N		389								
Mann–Whitney U		13,445,500								
Wilcoxon W		67,401,500								
Test Statistic		13,445,500								
Standard Error		711,752								
Standardized Test Statistic		4,835								
Asymptotic Sig.(2-sided test)		** < 0,001**								

### GROUP 2 (graduate dentists without post-graduate training/graduate dentists with postgraduate training in specializations other than oral surgery/graduate dentists with post-graduate training in oral surgery)

Results obtained from graduate dentists who had received different types of postgraduate training or no postgraduate training were compared by means of the Kruskal Wallis test for independent samples.

All the graduate dentists without postgraduate training believed that the clinician’s experience was the most influential factor, followed by anatomy, and radiographic factors, patient sex being the least important for all groups. At the same time, graduate dentists without postgraduate qualifications and those with postgraduate training other than oral surgery considered that operative factors were more influential than patient-related factors, giving lower scores to the latter with the exception of mouth opening. Conversely, dental graduates who had completed post-graduate programs in oral surgery placed more importance on patient-related factors (mouth opening, age, body mass index, ethnicity) than operative factors (with the exception of the clinician’s experience).

**Fig. 2:**
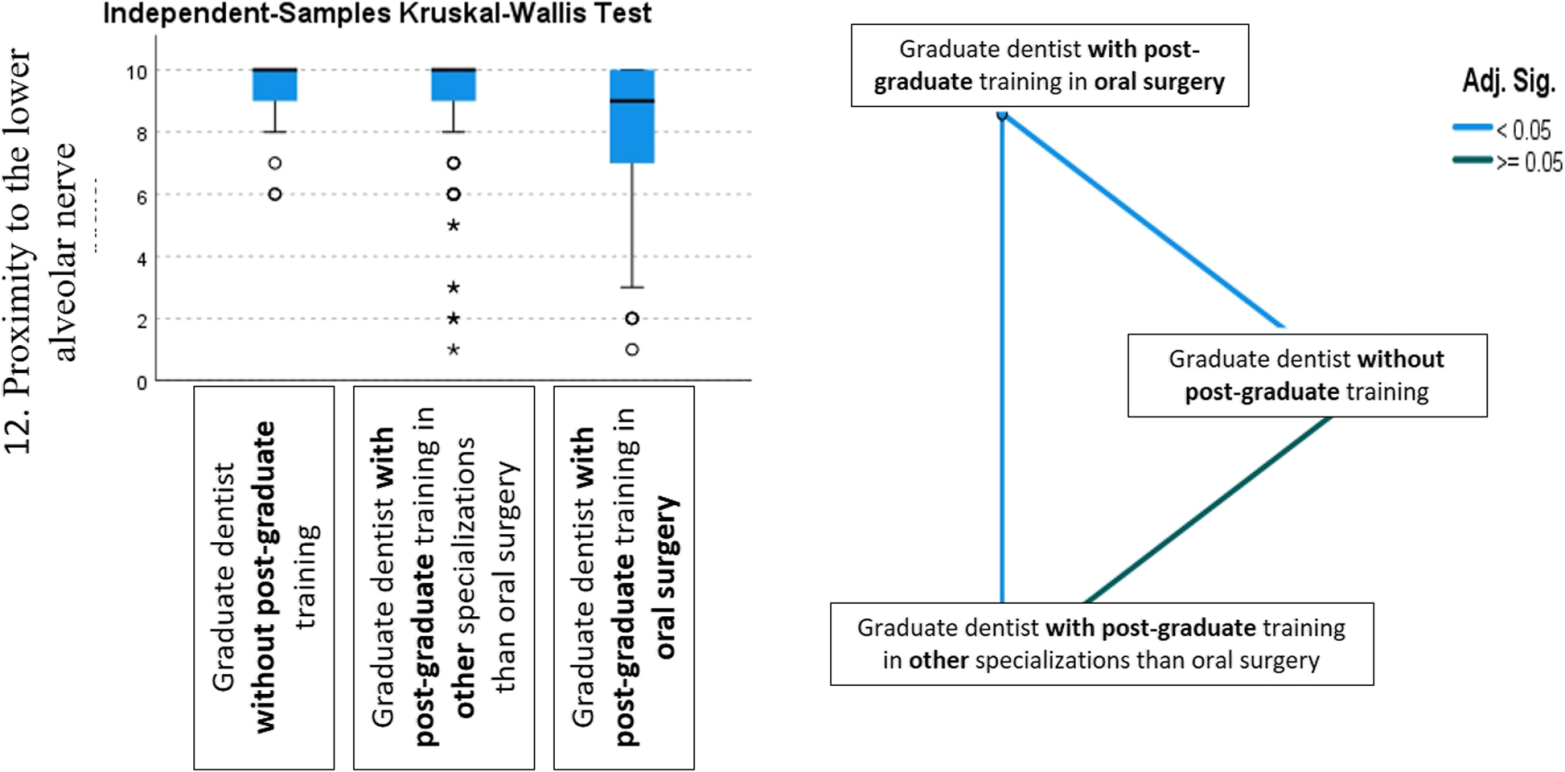
Boxplot and graphical representation of the pairwise comparison of the assessment of the factor "PROXIMITY TO INFERIOR ALVEOLAR NERVE" within Group 2. Note that dentists with postgraduate training in oral surgery considered this factor less important than dentists without training in oral surgery

Significant differences between groups were found (*p* < 0.05) for six out of the 17 factors. Two of these were demographic factors (age and ethnicity), given higher scores by dentists with post-graduate training in oral surgery. Another four factors were given higher scores by graduate dentists without postgraduate training, three of these being anatomical (situation, impaction depth, and proximity to the inferior alveolar nerve), and one operative (need for flap raising). The largest statistical difference was found for proximity to the inferior alveolar nerve, a factor that dentists with postgraduate training in oral surgery considered less important than dentists without training in oral surgery (*p* < 001) (Table 4 and Fig. 2).

**Table 4:** Values given by graduate dentists without post-graduate training/graduate dentists with postgraduate training in other specializations than oral surgery/graduate dentists with post-graduate training in oral surgery to the factor “PROXIMITY TO THE INFERIOR ALVEOLAR NERVE”. Krustal Wallis test and pairwise comparison

			Valid N	Mean		Standard Deviation		Median	Percentile 25	Percentile 75	Minimum		Maximum
Proximity to the Inferior alveolar nerve	Group 2	Graduate dentists **without post-graduate** training	33	9,30		1,19		10,00	9,00	10,00	6,0		10,0
		Graduate dentists with **postgraduate** training in **other** specializations than oral surgery	115	8,98		2,00		10,00	9,00	10,00	1,0		10,0
		Graduate dentists with **post-graduate** training in oral **surgery**	180	8,24		2,25		9,00	7,00	10,00	1,0		10,0
		Total	328	8,61		2,12		10,00	8,00	10,00	1,0		10,0
**Independent-Samples Kruskal–Wallis Test Summary**													
Total N			328										
Test Statistic			15,875^a^										
Degree Of Freedom			2										
Asymptotic Sig.(2-sided test)			** < 0,001**										
a. The test statistic is adjusted for ties													
**Pairwise Comparisons of Group 2**													
Sample 1-Sample 2			Test Statistic		Std. Error		Std. Test Statistic			Sig		Adj. Sig.^a^	
Graduate dentists with **post-graduate** training in oral **surgery-** Graduate dentists with **postgraduate** training in **other** specializations than oral surgery			37,013		10,315		3,588			< 0,001		0,001	
Graduate dentists with **post-graduate** training in oral **surgery**—Graduate dentists **without post-graduate** training			41,889		16,362		2,560			0,010		0,031	
Graduate dentists with **postgraduate** training in **other** specializations than oral surgery- Graduate dentists **without post-graduate** training			4,875		17,063		0,286			0,775		1,000	

Each row tests the null hypothesis that the Sample 1 and Sample 2 distributions are the same. Asymptotic significances (2-sided tests) are displayed. The significance level is 0,050

^a^Significance values have been adjusted by the Bonferroni correction for multiple tests

**Fig. 3:**
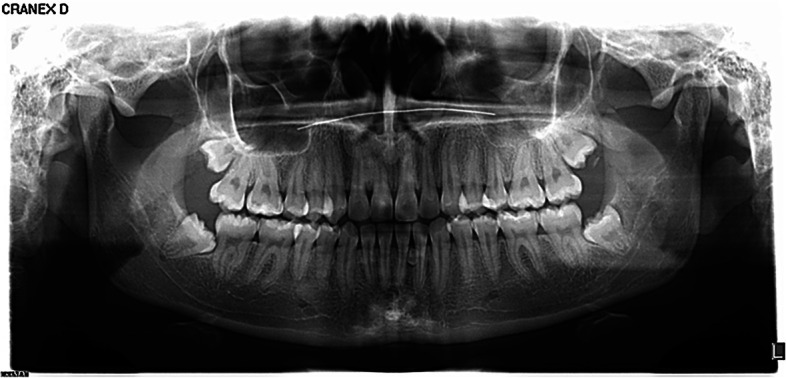
Panoramic radiograph number 3 used in the survey for evaluation of MTM 38

Comparing the perception of surgical difficulty of the 30 cases of mandibular third molars, only four cases exhibited no significant differences between groups of dentists. In the other 26 cases, statistical differences were found whereby dentists with postgraduate training in oral surgery gave lower scores than those without surgical training. (Fig. 3).

### GROUP 3 (graduate dentists who completed post-graduate programs in oral surgery of three years/two years/one year duration/modular course with non-weekly attendance).

Sub-groups were compared in order to find out whether the type of post-graduate program in oral training and its duration had any effect on perceptions of surgical difficulty. These were as follows: graduate dentists who had completed a postgraduate program in oral surgery of three years duration, two years duration, one year duration or a modular course without weekly attendance. The results for the four sub-groups were compared by means of the Kruskal Wallis test for independent samples, applying paired comparisons whenever the results showed significant differences (*p* < 0.05).

The one factor that obtained significant differences between sub-groups (*p* < 0.05) was mandibular third molar proximity to the inferior alveolar nerve, whereby dentists who had received training of the shortest duration gave this factor a higher score (9.7 +—0.7) in comparison with those who had undergone training of longer duration (7.3 +—2.3).

Dentists who had completed modular courses in oral surgery gave this factor the greatest importance, while the other three sub-groups assessed the clinician’s experience as the most influential factor.

**Fig. 4:**
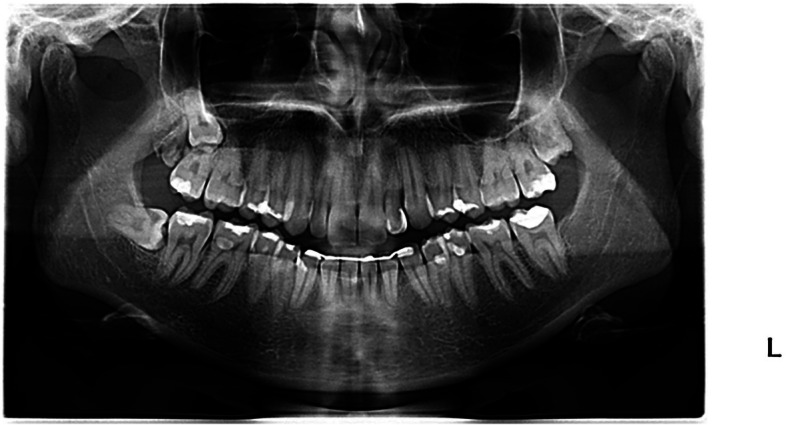
Panoramic radiograph number 9 used in the survey for evaluation of MTM 48

Significant differences (*p* < 0.05) were found in four of the 30 cases assessed in terms of surgical difficulty, in which the highest values were given by those who had completed modular courses (Fig. 4). For the other 26 cases there was a tendency for dentists who had undergone training of shorter duration to award higher scores, although this did not reach statistical significance.

## Discussion

Determining the potential difficulty of MTM extraction surgery pre-operatively is essential for correct surgical planning and helps to minimize risk and avoid possible intra- and post-operative complications.

Several researchers have assessed dentists’ ability to predict surgical difficulty, obtaining widely differing results [15, 23, 25–29]. These studies used diverse assesment methods, complicating any comparison between previous studies and the present work. In addition, the present study investigated perceived difficulty rather than predicted difficulty (which would involve actual extractions afterwards), assessing whether training and specialization affected dentists’ perceptions of surgical difficulty.

Values given to the perceived difficulty of MTM surgery were lower among dentists with postgraduate training in oral surgery with statistically significant difference. This finding concurs with results published by Barreiro-Torres et al. [23], who found greater predictive ability among oral surgeons than general dentists. This could be due to better training in foreseeing the right technique to use and more learning about those factors that affect the complexity of the procedure. In the same way, McCluskey et al. [29] observed a greater predictive capacity among oral surgeons compared with dental undergraduates.

In the present work, 52.4% of undergraduates stated that they had received no training in this respect. It is remarkable that 18.9% of graduate dentists who had undergone no postgraduate training in oral surgery also reported having received no training in this field. In addition, 2.2% of dentists with post-graduate training in oral surgery reported not having received any training, these being dentists who had completed modular courses in oral surgery with no weekly attendance. This points to a need to review the syllabuses of these programs so that adequate information is imparted to all dentists. Given the importance of assessing sugical difficulty pre-operatively in order to ensure safe treatment and minimum risk, dentists need sufficient information to know when it is necessary to refer the patient to another better qualified surgeon.

The present work also set out to establish which variables – patient-related, anatomical/radiographic, or operative – were considerd to have the most influence on surgical difficulty. Other researchers report that oral surgeons believe anatomical factors to be the most influential, followed by operative factors, while patient-related (demographic) factors are allotted the least importance [15]. But in the present study, dentists with postgraduate training in oral surgery gave greater importance to patient-related factors than operative factors, with the exception of the clinician’s experience, which they thought the most important. These findings concur with Akadiri [6] and Susarla [15]. This contrasts with results obtained for dental undergraduates and graduate dentists without prostgraduate training in oral surgery, who considered operative factors more influential than patient-related factors. This difference could be due to the skills acquired by those with specialized training in oral surgery, such as flap raising, performing ostectomies or odontosections, and so on, as once the techniques and skills have been learnt, they will not complicate the procedure or present technical challenges, and may even facilitate surgery. At the same time, oral surgeons may have greater awareness of other factors that influence surgical difficulty. In a similar vein, Susarla and Dodson [15] stress that it is precisely the fact of not taking patient-related factors into account (age, sex, ethnicity, body masss index, mouth opening) that leads to inadequte pre-operative assessments of surgical difficulty. Therefore, it is essential to insist on the transcendence of these other factors when it comes to training students in MTM extraction.

Regarding anatomical factors, it should be noted that participants in the present survey who had not received post-graduate surgical training and those with surgical training of short duration (modular courses) gave greater importance to the proximity of the MTM to the inferior alveolar nerve, while for those specialized in oral surgery this factor was the least influential. Nevertheless, this factor can influence the duration of surgery. In this sense, Alvira-Gonzalez et al. [18] and Sánchez-Torres et al. [17] found that MTM close to the alveolar nerve required longer surgical time due to the careful attention needed to avoid damaging the nerve [18].

According to the present findings, dentists with specialized training in oral surgery considered that the most influential operative factor (apart from the surgeon’s experience) was the need to perform odontosection, a factor that other researchers have also cited as signficiantly related to surgical difficulty [17, 18].

Differences found between dentists with postgraduate training in oral surgery and those without highlighted the different levels of knowledge and experience among these groups. This is bound to have repercussions when it comes to performing MTM extractions.

The present work points the way forward to future research to determine how the perceived difficulty of MTM extraction – clearly related to the individual dentist’s level of training and specialization – corresponds to data obtained once the third molar has been extracted. In the same way, it would be interesting and useful to conduct similar surveys of other interventions in the field of oral surgery and implant dentistry, comparing both the perceived difficulty of these interventions with final treatment outcomes in relation to the dental training received.

The limitations of the present study include the different numbers of participants in each group – oral surgeons being the most represented – and the fact that the population studied was limited to a subgroup of the Spanish population (located in Madrid). It would therefore be interesting to carry out futher studies to compare different subgroups of the Spanish population or even to compare training according to different countries.

## Conclusions

Despite the limitations of this study, it may be concluded that dental training has a signficant influence on the perceived difficulty of MTM extraction, whereby dentists with no surgical training see the intervention as presenting greater difficulty on visual analogue scales. In the same way, specialized training in oral surgery signficantly affects opinions about which factors have more or less influence on surgical difficulty. Professionals with postgraduate training in oral surgery considered patient-related factors more important than operative factors, in contrast to dentists who had not received oral surgery training, who believed operative factors to have more influence on levels of surgical difficulty.

## Supplementary Information

**Additional file 1. **

## Data Availability

Databases used and/or analyzed during the current study are available from the corresponding author upon reasonable request.
