# Supplementary material for: Mandibular third molar extraction: perceived surgical difficulty in relation to professional training
Source: BMC Oral Health. 2023 Jul 14;23:485. doi: 10.1186/s12903-023-03131-7 (PMC10349451; doi:10.1186/s12903-023-03131-7)
Supplement: Supplementary file 1 — Additional file 1. [file 12903_2023_3131_MOESM1_ESM.docx]

|  |  |
| --- | --- |

**Perception of the surgical difficulty of lower third molar extractions**

**Extraction of the lower third molar is one of the most frequent interventions of the oral surgeon. It is a laborious intervention that requires experience.**
**This questionnaire is completely anonymous and aims to assess the perception of the difficulty of surgery of the third lower molar among dental practitioners according to their level of experience and training.**
**Their involvement will be very useful. Many thanks for your help.**

I agree to participate in this anonymous questionnaire and to have my answers analysed for research purposes

Yes I consent

I do not consent

Thank you very much for your time.
Survey ended

**Demographic and training data**

1.Please indicate the age group in which you are:

|  | Answer  18-22 years old |
| --- | --- |
|  | Answer  23-27 years old |
|  | Answer  28-30 years old |
|  | Answer  31-35 years old |
|  | Answer  36-45 years old |
|  | Answer  46 or more than 46 years |

***** Question2.Please indicate your gender

|  | Answer  Male |
| --- | --- |
|  | Answer  Female |
|  | Answer  Other |

***** Question3.Please indicate the situation in which you find yourself

|  | Answer  I am a dental student |
| --- | --- |
|  | Answer  I have finished my dental career |
|  | Answer  I am a maxillofacial surgeon or I am living   Question4. Year in which the dental career ended   \|  \| Answer  Before 1995 \| \| --- \| --- \| \|  \| Answer  Between 1996 and 2005 \| \|  \| Answer  Between 2006 and 2010 \| \|  \| Answer  Between 2011 and 2015 \| \|  \| Answer  Between 2016 and 2021 \| |

***** Question5. When do you complete your maxillofacial surgery studies?

|  | Answer  I am living |
| --- | --- |
|  | Answer  I completed residence between 2016 and 2021 |
|  | Answer  I completed residence between 2011 and 2015 |
|  | Answer  I completed residence between 2010 and 2006 |
|  | Answer  I completed residence between 2005 and 1996 |
|  | Answer  I completed residence before 1995 |

***** Question6. Have you completed or are you doing any postgraduate degree in dentistry?

|  | Answer  Yes |
| --- | --- |
|  | Answer  No |

***** Question7. what type of postgraduate have you done or are you doing? You can tick several boxes.

|  | Answer  a. Oral surgery |
| --- | --- |
|  | Answer  B. Implantology |
|  | Answer  B. Periodontics |
|  | Answer  C. Orthodontics |
|  | Answer  D. Prosthodontics |
|  | Answer  e. Endodontics |
|  | Answer  f. Other |

8.If you have completed or are undergoing a postgraduate degree in oral surgery, please write at which university or school you completed


***** Question9.If you completed or are performing a postgraduate degree in oral surgery, when did you complete it?

|  | Answer  a. I am not performing any postgraduate degree in oral surgery |
| --- | --- |
|  | Answer  b. I am in the first course |
|  | Answer  c. I am in second course |
|  | Answer  D. I am in the third course |
|  | Answer  e. I finished less than 5 years ago |
|  | Answer  f. I finished between 5 and 10 years ago |
|  | Answer  g. I finished more than 10 years ago |

***** Question10.That postgraduate in surgery had a duration of:

|  | Answer  a. Master’s 3 years of weekly attendance |
| --- | --- |
|  | Answer  b. Master’s 2 years of weekly attendance |
|  | Answer  C. 1 year attendance course per week |
|  | Answer  D. Modular non-weekly attendance course |
|  | Answer  e. I am not performing any postgraduate degree in oral surgery |

Surgical difficulty

***** Question11. do you think you are aware of the factors that influence the surgical difficulty of extracting the lower third molars?

|  | Answer  Yes |
| --- | --- |
|  | Answer  No |

***** Question12. have you been trained on the factors influencing the surgical difficulty of extracting the lower third molars?

|  | Answer  Yes |
| --- | --- |
|  | Answer  No |

**Predictive variables of surgical difficulty.**
Below are different variables that may be related to the difficulty of extracting the lower third molar
Please indicate on the scale from 0 to 10 the influence that this factor represents on the surgical difficulty, with 0 being the lowest and 10 being the highest.

Minor importance

Most important

0

10

- Question1.Patient’s sex
- Question2.Patient’s age
- Question3.Patient’s Ethnicity
- Question4. Mouth opening of the patient
- Question5.Patient’s body mass index
- Question6.Third molar root morphology
- Question7.Third molar root curvature
- Question8.Position of the third molar (inclination: mesioangular, distoangular, horizontal...)
- Question9. Situation of the third molar (erupted, impacted, including)
- Question10. depth of impaction
- Question11. impaction of lower third molar in the ascending ramus
- Question12. Proximity of the lower third molar to Inferior alveolar nerve
- Question13. anesthetic technique: local vs. general anesthesia
- Question14. need for raising a flap
- Question15. need for ostectomy
- Question16. need for odontosection
- Question17. clinician’s experience

**Radiographic images**.
Below there are a series of panoramic X-rays. Please note on the scale from 0 to 10 the difficulty you consider to be present in the extraction of the cordal indicated, 0 being the absence of difficulty and 10 being the maximum difficulty.

1. Extraction of 48

Minimal difficulty

Maximum difficulty

0

10

(2-30 Thirty panoramic X-rays are shown)

The survey has been finalised.
Thank you for your trust and collaboration
